# Lung cancer presenting as a metastasis to the carpal bones: a case report

**DOI:** 10.1186/1752-1947-6-384

**Published:** 2012-11-13

**Authors:** Giuseppe Rinonapoli, Auro Caraffa, Renato Antenucci

**Affiliations:** 1 Orthopaedic Department, University of Perugia, Perugia, Italy; 2 S. Andrea delle Fratte, Perugia, 06100, Italy

## Abstract

**Introduction:**

A first metastasis to the hand is extremely rare. Usually, an acrometastasis is a sign of very advanced disease, with the presence of previous multiple metastases elsewhere. The present paper is one of the very few case reports of first metastatic location to carpal bones. To date, only Lederer *et al*., in 1990, and Song and Yao in 2012, have described a metastasis to the trapezium from lung cancer.

**Case presentation:**

A 74-year-old Caucasian man was submitted to several physical examinations for thumb pain. The first diagnosis was tendonitis and the second diagnosis was thumb carpometacarpal osteoarthritis. Only when the patient was admitted to an internal medicine department for deterioration of his general condition and an enormous mass on his left hand was an open biopsy performed. It revealed a metastasis from large-cell lung carcinoma. A total-body scintigraphy and total-body computed tomography scan were negative for other secondary locations. The patient underwent an amputation at the distal third of the forearm.

**Conclusion:**

Less than 20 case reports are available in the literature dealing with metastases to carpal bones. Very few cases are described as carpal metastases in the absence of other previous metastases, and only two articles, before the present one, have reported a metastasis to the trapezium. This case report teaches us two things: first, patient adherence to follow-up is extremely important; and, second, a thorough examination of diagnostic findings needs to be carried out at all times.

## Introduction

More than 50% of cancers disseminate to the skeleton, which is the third most frequent site of metastatic spread after the lung and the liver [1]. Epidemiological investigation has shown that, of the 1.2 million new cases of cancer each year in the USA, about 300,000 will eventually develop a bone metastasis [2]. Bone metastasis is frequently seen in patients with breast cancer and prostate cancer, with up to 67% to 75% of patients developing bone metastases. The incidence of bone metastases in post-mortem examination in lung cancer is about 30% [3]. The sites most involved, in order of frequency, are: spine, pelvis, ribs, skull and proximal long bones [2].

The upper limb of the skeleton is least affected by bone metastases with approximately 10% to 15% occurring in this region [4].

The case described in the present paper is relative to a delayed diagnosis of a metastasis to the carpal trapezium from lung cancer.

## Case presentation

A 74-year-old Caucasian man, who was a regular drinker and heavy cigarette smoker, presented to a rheumatologist for pain at motion of his left thumb. The physician diagnosed a tendonitis, and the patient was prescribed anti-inflammatory drugs, which allowed for a moderate improvement in the pain.

The patient presented one month later to our clinic complaining of persistent pain in his left hand, even at rest. A physical examination revealed a severe tenderness of the first metacarpophalangeal joint and, though less severe, of the lateral carpal region. Pain increased with the mobilization of the wrist and the thumb.

**Figure 1:**
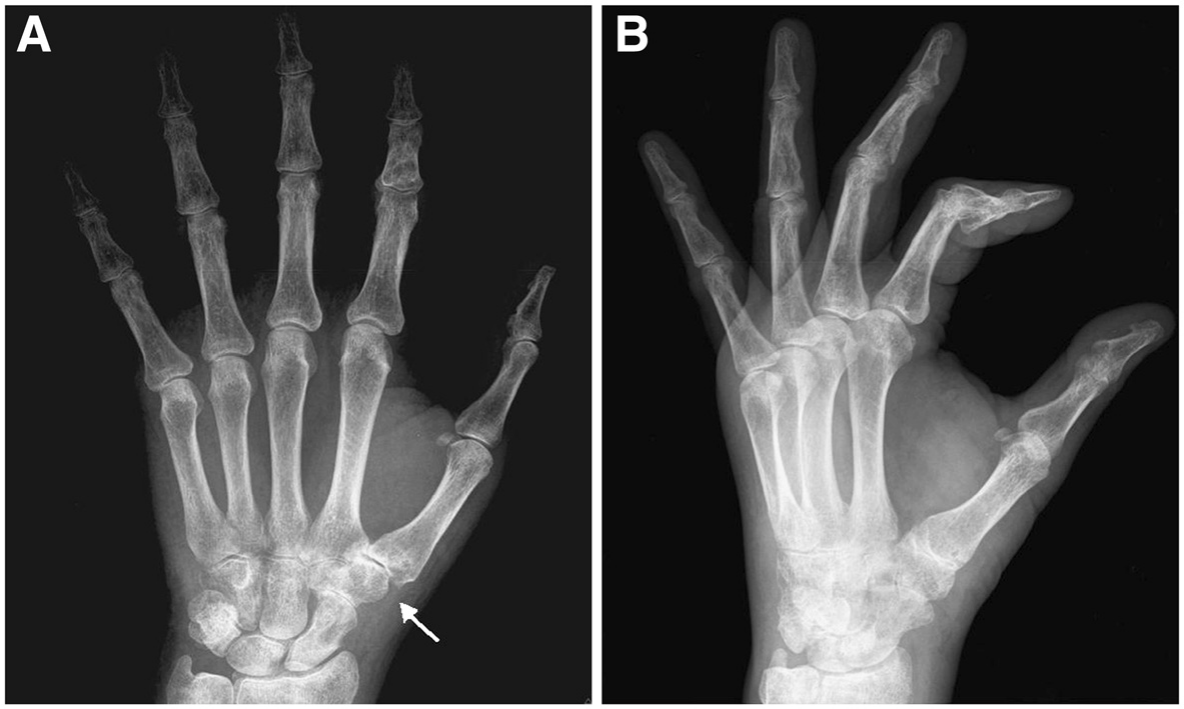
**Plain radiograph of the hand performed after the second clinical examination.** (**A**) Anteroposterior view. (**B**) Lateral view. Space narrowing of the trapeziometacarpal and trapezionavicular joints (*arrow in *[**A**]). Diffuse osteoporotic picture of the carpal and the phalangeal bones, to be interpreted as disuse osteoporosis.

The patient was prescribed plain radiographs of his left hand which showed diffuse osteoporosis and signs of osteoarthritis of the trapeziometacarpal joint (Figure 1). A diagnosis of thumb carpometacarpal osteoarthritis was made. A hand surgeon prescribed a specific brace.

**Figure 2:**
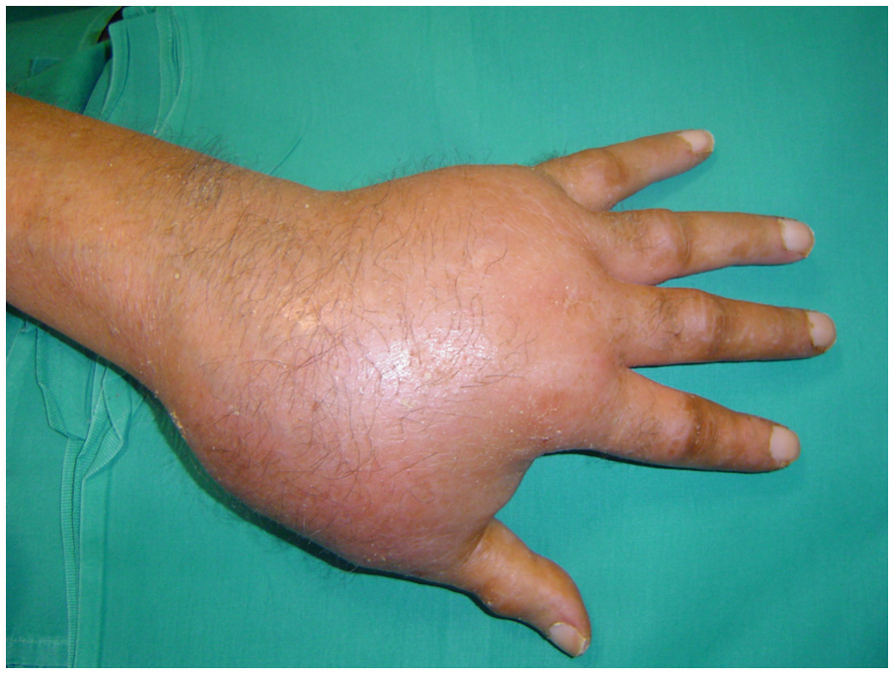
Large mass involving the whole hand.

**Figure 3:**
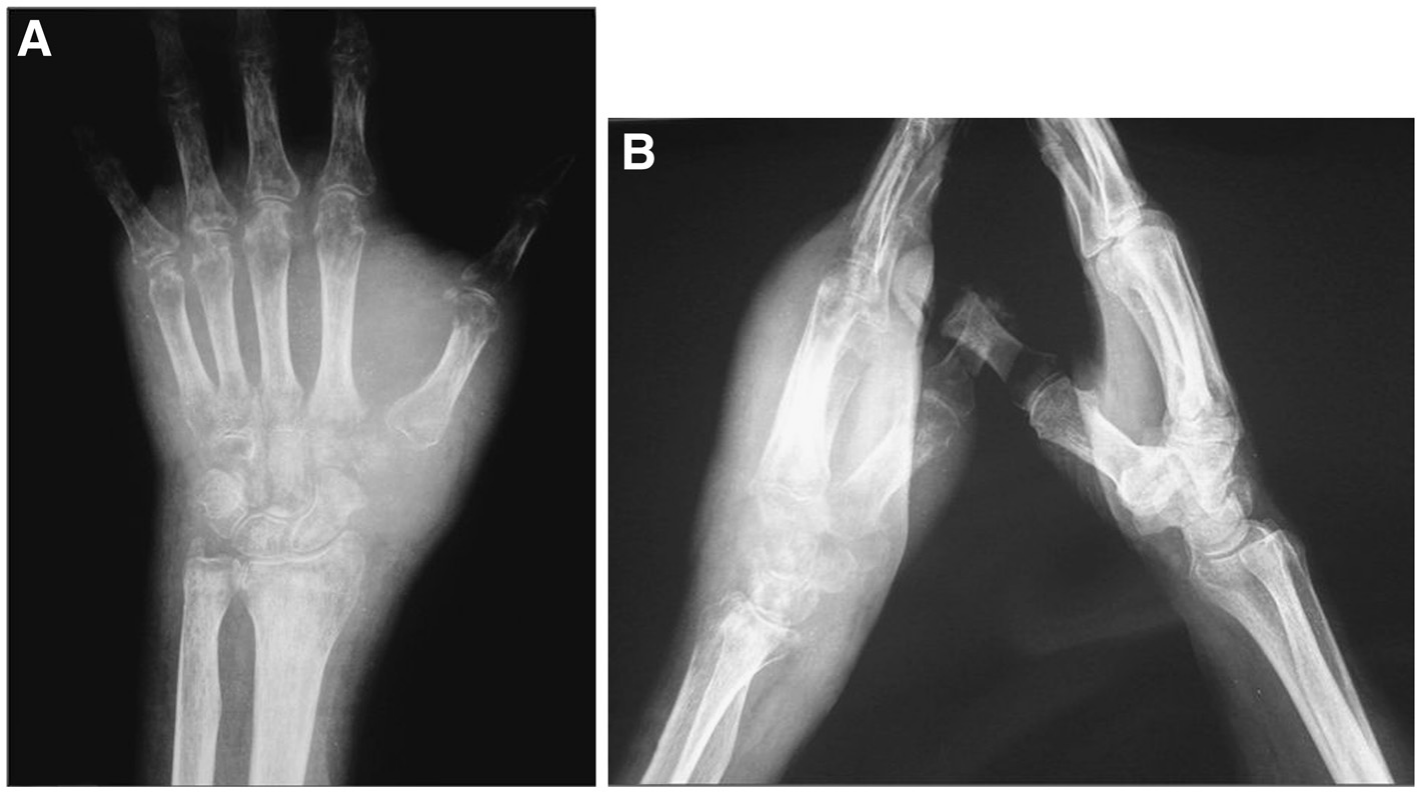
**Plain radiograph of the left hand: almost total resorption of the trapezium and trapezoid.** Diffuse osteoporosis. (**A**) Anteroposterior view. (**B**) Lateral view of both hands.

**Figure 4:**
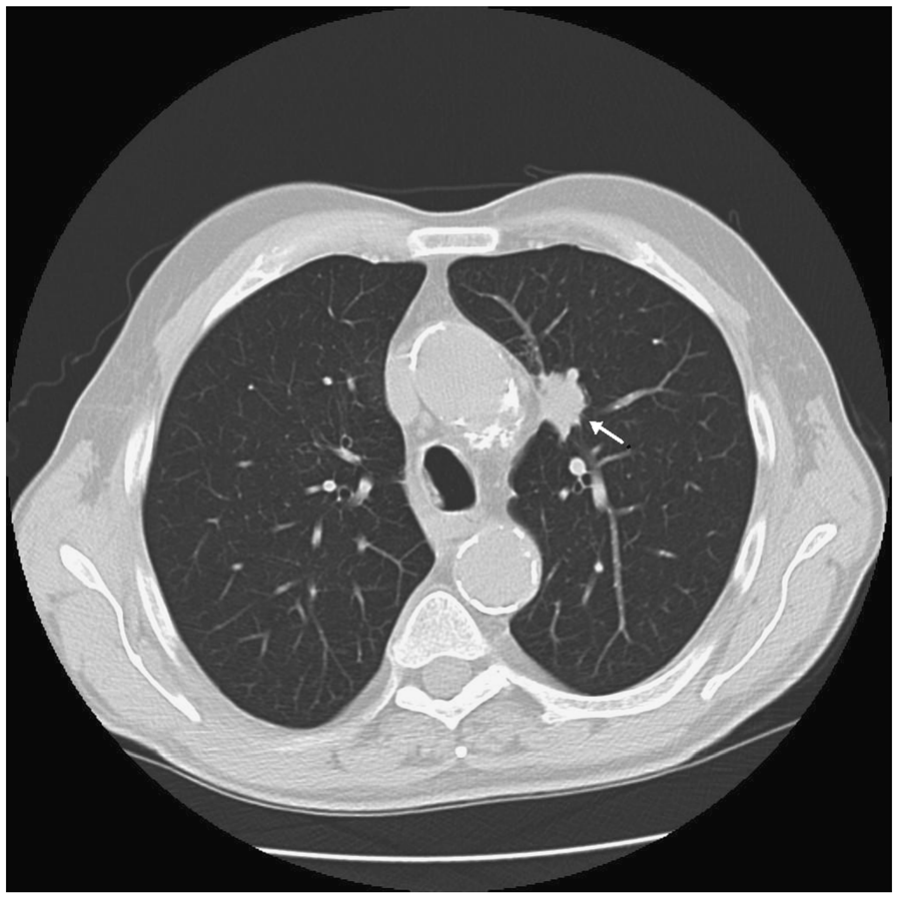
Chest computed tomography scan revealing a mass in the superior left lobe (arrow).

We had no contact with the patient after his follow-up, which was held two months after his presentation to our clinic, until he was admitted to the internal medicine department of our hospital three months later for severe asthenia and a weight loss of 10kg over the previous five months. The patient declared to have been eating normally until the last month when his appetite was reduced. The patient was examined by one of the authors of this report for an enormous mass that involved his whole left hand (Figure 2). Severe pain discouraged the patient from using his left hand in daily activities. Pain was even present on raising the hand from a table. Physical examination showed a large mass of his left hand, deep tenderness, an increase in local temperature and edema. Simply touching the hand caused excruciating pain. No palpable lymph nodes were present. The results of laboratory tests showed: an alkaline phosphatase of 408mg/dL, erythrocyte sedimentation rate, leukocytes, C-reactive protein, antistreptolysin O, rheumatoid factor, immunoglobulin G (IgG), IgA, IgM, C3, C4, circulating immune complexes, antinuclear antibodies, antimitochondrial antibodies, carcinoembryonic antigens, α-fetoprotein and prostate-specific antigen were normal. Calcemia was also in the limits of normality. An ecography of the hand revealed ‘synovial hypertrophy, with rich vascularisation’. The patient received a new plain X-ray of his hand, which revealed a diffuse osteoporosis of the whole hand, with resorption of almost the whole trapezium, about 50% of the trapezoid, and erosion of the distal part of the scaphoid. The metacarpal and phalangeal bones were severely osteoporotic (Figure 3). A chest X-ray induced the suspicion of a malignant lesion in the superior lobe of the patient’s left lung. A subsequent computed tomography (CT) scan of the chest showed a large mass in that area (Figure 4).

**Figure 5:**
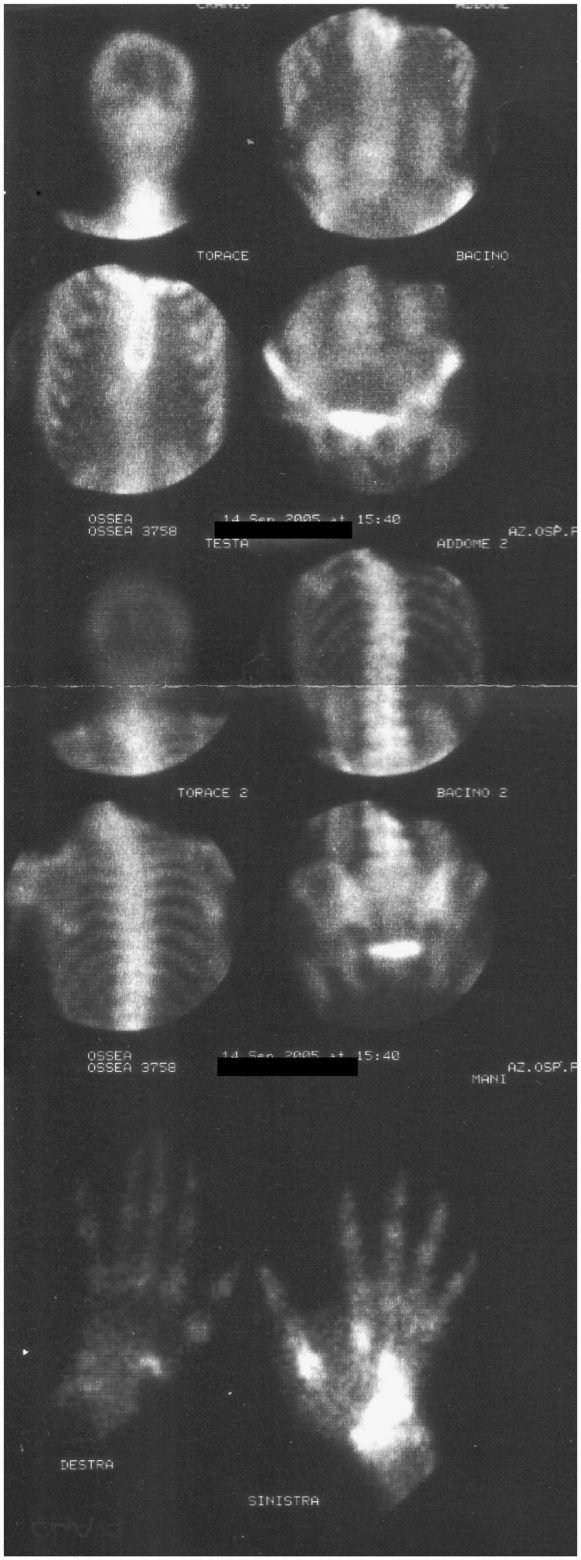
**Total-body bone scan with Tc**^**99**^**: unique hot area in correspondence of the left hand.**

At this point, the patient received a total-body bone scan with Tc^99^, which revealed the presence of a unique hot area corresponding to the left hand (Figure 5). A total-body CT scan was negative for other locations.

**Figure 6:**
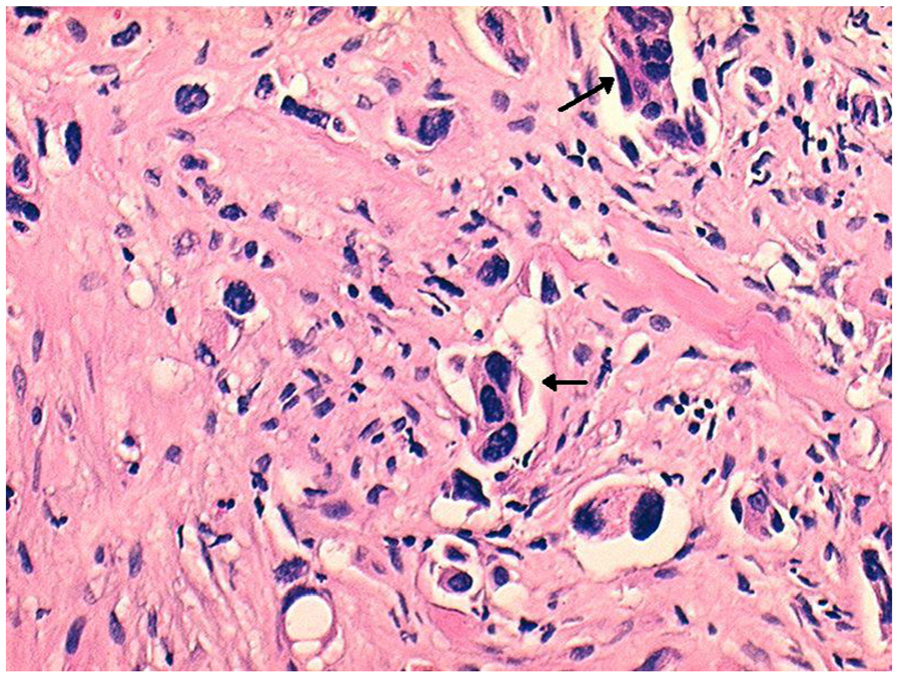
**Histology of the biopsy sample.** Clusters of anaplastic cells with numerous nucleus atypia (*arrows*) are observable (hematoxylin and eosin staining, magnification 200×).

**Figure 7:**
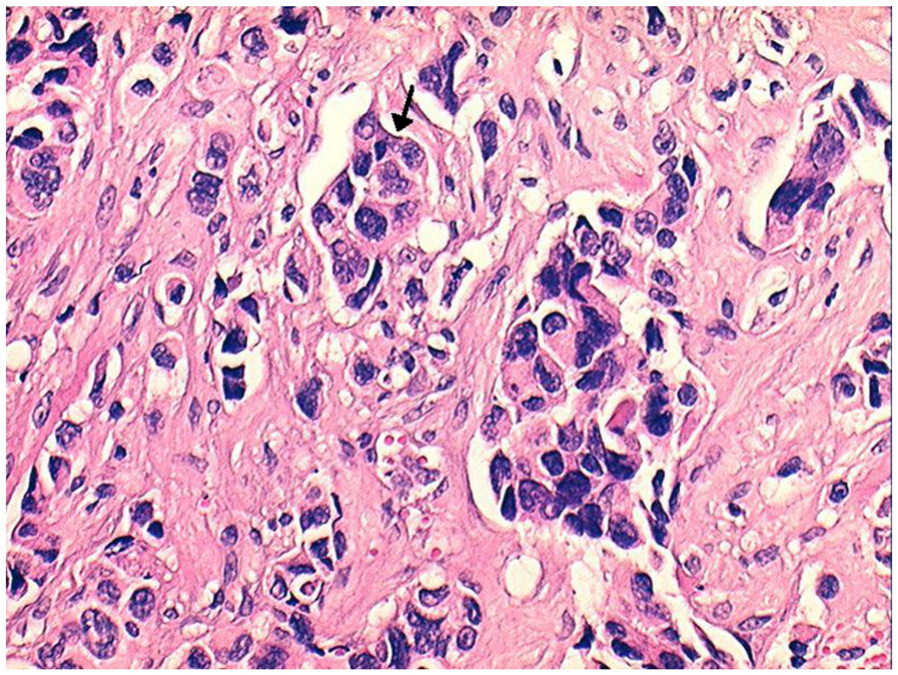
**Histology of the biopsy sample.** Clusters of anaplastic cells (*arrow*) are present in the lymphatic vessels (hematoxylin and eosin staining, magnification 200×).

**Figure 8:**
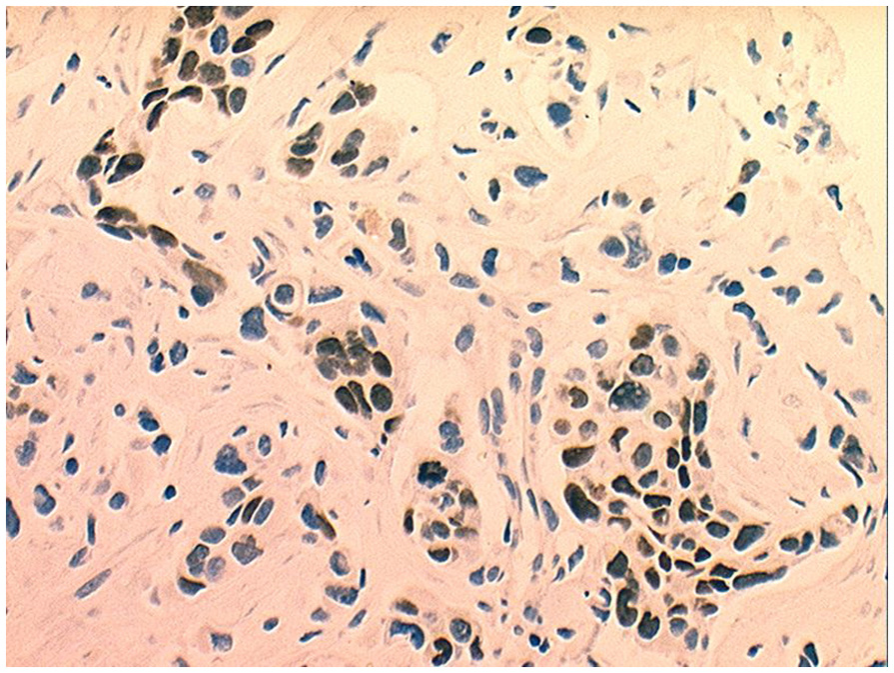
**Thyroid transcription factor-1 is expression of lung metastatic tumors.** The picture is positive for large-cell lung carcinoma.

The patient underwent an open biopsy. Histology revealed an undifferentiated large-cell carcinoma (Figure 6). Immunohistochemistry was thyroid transcription factor-1 positive, which was a confirmation of the lung origin (Figures 7 and 8). The diagnosis was metastasis of undifferentiated large-cell lung carcinoma. Magnetic resonance imaging (MRI) was attempted twice without success because the patient could not tolerate to keep his left hand steady.

We performed an amputation at the distal third of the forearm.

At six-month follow-up, the amputated forearm was in perfect condition. The patient was submitted to chemotherapy but, at present, his general condition is worsening because of respiratory problems.

## Discussion

The upper limb is the part of the skeleton least affected by bone metastases with approximately 10% to 15% of bone metastases occurring in this region. The primary tumors that more often metastasize here are cancers of the breast (73%), lung (32%), and kidney (24%) [5]. Furthermore, bone metastases located below the elbow or the knee are very uncommon, representing barely 4.1% of affected skeletal sites [6].

The carpal lesion described in the present paper was initially mistaken for a non-neoplastic lesion. One of the reasons for the delayed diagnosis was probably the rarity of this neoplastic site. The first location of the metastasis was probably the trapezium, although we cannot exclude the previous involvement of the trapezoid bone. In fact, the first X-rays showed signs of remodeling of the trapezium, but we cannot be certain that the trapezoid was lesion-free. The first radiographs (Figure 1) could have led to a diagnosis different from trapeziometacarpal osteoarthritis. In fact, if we carefully examine the anteroposterior view, we can notice that the space narrowing is more accentuated at the joint between the trapezoid and trapezium and between those bones and the second metacarpal bone than between the trapezium and the first metatarsal bone. This is a rather anomalous finding. The following radiographs (Figure 3) were made at an advanced stage of the disease: the trapezium appeared more widely resorbed than the trapezoid. This suggests the involvement of the trapezium as a first location.

A recent review of hand metastases [7] reported 257 cases of acrometastases to the hand; however, this review excluded carpal bones. In a review on hand metastatic diseases [8], Kerin reported that only about 0.1% of primary tumors metastasize to the hand, but only one case occurred in the carpus. Case reports on metastases to carpal bones are very few [9,10]. The article by Lederer *et al*. [11], published in *Radiologe* by a group of German radiologists, and the recent article by Song and Yao [12] are the only ones that have described a metastasis to the trapezium from a lung cancer.

The rarity of a malignant tumor located in the hand can induce a physician to misinterpret a lesion visible at the X-rays, and even more so a metastasis, which is even rarer in that region. Metastases to the carpal bones are much rarer than those to the metacarpal and phalangeal bones. For this reason, a metastasis in the carpus is more often misdiagnosed.

The described case regards a metastasis from severely undifferentiated large-cell lung carcinoma. Large cell carcinoma is classified among the non-small-cell lung carcinomas (NSCLCs), which comprise a heterogeneous group of histology types, with the most frequent types being adenocarcinoma, squamous cell carcinoma, large cell carcinoma, adenosquamous carcinoma and sarcomatoid carcinoma. Large cell carcinomas account for approximately 9% of all lung cancers [13]. Large cell carcinoma tends to metastasize to the brain (50% of the cases), mediastinal lymph nodes, bones and the liver [14]. Stage IV NSCLC is an incurable disease [15].

The first and the second clinical examinations of the patient’s hand induced the physicians to make the most likely diagnosis: tendonitis (first physician), thumb osteoarthritis (second physician); both of which are very common pathologies. The first physician based his diagnosis on clinical symptoms (pain at motion of the left thumb, not at rest), probably waiting upon the results of his therapy. One month later, the second physician made his diagnosis on the basis of the patient’s age, the clinical picture, which included prevalent pain and tenderness located at the first metacarpophalangeal joint, and the radiological picture. The combination of the clinical and radiological pictures could actually induce the suspicion of thumb osteoarthritis. The radiological picture of diffuse osteoporosis led the physician to think of a diffused disuse osteoporosis, caused by the severe pain.

When the patient presented to the third physician it was an emergency admission to an internal medicine department because progressive worsening of his general condition was evident as well as an enormous swelling of the left hand. The patient’s compliance was undoubtedly poor. The correct diagnosis would have been made sooner if the patient had presented at the requested follow-ups.

An early diagnosis was not easy, but the first two physicians examining the patient might have been too superficial in their anamnesis, clinical examinations and X-ray interpretations. If the second physician had better investigated the patient’s general conditions, he would have discovered that the patient had already lost 10kg without a clear cause. One more clue should have been the fact that the patient was a heavy cigarette smoker. These facts should have made the two physicians suspicious. Moreover, the second physician underestimated the radiological signs of diffuse osteoporosis which were probably, in part, areas of osteolysis.

It was probably not logical to imagine a malignant location on the basis of the data that arose from the first clinical examinations and X-rays. The rarity of metastases to the distal regions of the limbs can induce a physician to suspect a primary tumor of the hand. They are rare, too, especially in the carpal bones. When a metastasis to the hand is discovered, it is very probable that other (previous) metastases are disseminated in the skeleton, because metastases of hand bones generally indicate very advanced primary tumors. The hand as the first location of metastasis is the main peculiarity of the presented case. Metastases to the hand are rare, but those to the carpal bones are even rarer. The location of the lesion at the trapezium, as the first X-rays showed, led the examining physicians to suspect other disorders.

## Conclusion

The described case was not easy to diagnose because of the poor compliance of the patient and the rarity of carpal bone metastasis. This case report teaches us two things: first, patient adherence to follow-up is extremely important; and, second, a thorough examination of diagnostic findings needs to be carried out at all times.

## Consent

Written informed consent was obtained from the patient for the publication of this case report and its accompanying images. A copy of the written consent is available for review by the Editor-in-Chief of this journal.

## Competing interests

The authors declare that they have no competing interests.

## Authors' contributions

GR operated on the patient and was a major contributor in writing the manuscript. AC interpreted the patient's histological data. RA collected all the data in the anamnesis of the patient, and contacted the colleagues from Oncology, Pathology and Chest Surgery, to receive a multidisciplinary opinion about the case. All authors read and approved the final manuscript.
